# Opposite Self-Folding Behavior of Polymeric Photoresponsive Actuators Enabled by a Molecular Approach

**DOI:** 10.3390/polym11101644

**Published:** 2019-10-10

**Authors:** Daniele Martella, Sara Nocentini, Diego Antonioli, Michele Laus, Diederik S. Wiersma, Camilla Parmeggiani

**Affiliations:** 1 European Laboratory for Non-Linear Spectroscopy (LENS), via Nello Carrara 1, 50019 Sesto Fiorentino, Italy; nocentini@lens.unifi.it (S.N.); wiersma@lens.unifi.it (D.S.W.); camilla.parmeggiani@lens.unifi.it (C.P.); 2 National Institute of Optics, CNR-INO, via Nello Carrara 1, 50019 Sesto Fiorentino, Italy; 3 Dipartimento di Scienze e Innovazione Tecnologica (DISIT), Universitá del Piemonte Orientale “A. Avogadro”, INSTM, UdR Alessandria, Viale T. Michel 11, 15121 Alessandria, Italy; diego.antonioli@uniupo.it (D.A.); michele.laus@uniupo.it (M.L.); 4 Department of Physics and Astrophysics, University of Florence, Via G. Sansone 1, 50019 Sesto Fiorentino, Italy; 5 Istituto Nazionale di Ricerca Metrologica INRiM, Strada delle Cacce, 91, 10135 Turin, Italy; 6 Department of Chemistry “Ugo Schiff”, University of Florence, via N. Carrara 3-13, 50019 Sesto Fiorentino, Italy

**Keywords:** liquid crystalline networks, photoresponsive polymers, shape-changing materials, curvature design, photonic actuators

## Abstract

The ability to obtain 3D polymeric objects by a 2D-to-3D shape-shifting method is very appealing for polymer integration with different materials, from metals in electronic devices to cells in biological studies. Such functional reshaping can be achieved through self-folding driven by a strain pattern designed into the molecular network. Among polymeric materials, liquid crystalline networks (LCNs) present an anisotropic molecular structure that can be exploited to tailor internal strain, resulting in a natural non-planar geometry when prepared in the form of flat films. In this article, we analyze the influence of different molecular parameters of the monomers on the spontaneous shape of the polymeric films and their deformation under different stimuli, such as heating or light irradiation. Modifying the alkilic chains of the crosslinkers is a simple and highly effective way to increase the temperature sensitivity of the final actuator, while modifying ester orientation on the aromatic core interestingly acts on the bending direction. Combining such effects, we have demonstrated that LCN stripes made of different monomeric mixtures originate complex non-symmetric deformation under light activation, thus opening up new applications in photonic and robotics.

## 1. Introduction

Several techniques enable the shaping of polymers into 3D objects with tailored chemical-physical properties. While the exponential growth of 3D printing provides access to devices across different length scales (from nanometric to macroscopic scale) [1,2], indirect 3D self-folding methods introduce simple ways to integrate polymeric scaffolds with other components. These methodologies employ well-known planar fabrication technologies on deformable substrates that, after being realized as flat film, self-fold into a 3D fashion [3]. “2D processing for 3D functionalities” has been successfully exploited in biomedical engineering, where cells can be easily seeded on flat scaffolds that, after adhesion, can self-fold into a tubular shape, thus mimicking a blood vessel structure [4]. Another interesting application is the integration of electronic and optoelectronic microelements on non-planar surfaces. Micro silicon arrays fabricated in a standard planar layout can be integrated in a stretched, soft substrate that, after release, gives rise to a hemispherical geometry that can be employed for bioinspired electronic eye cameras [5].

Among different 2D-to-3D shape-shifting methods, the most common techniques are based on bilayer structures or on single materials characterized by a transversal gradient of the crosslinking degree. In both cases, the material anisotropy generates an asymmetric swelling in a solvent that forces a specific folding [6,7].

A different strategy to induce 3D self-folding relies on programming the molecular alignment inside liquid crystalline networks (LCNs), thus creating specific internal stresses that drive flat polymer reshaping, both in dry and liquid environments [8]. These polymers, well known for their shape-changing properties under external stimuli, are prepared by covalently bonding mesogenic units inside a polymeric network to obtain a highly anisotropic backbone [9,10]. At the phase transition (or when the partial disordering of liquid crystalline molecular arrangement occurs), a macroscopic deformation of the whole sample is achieved by a specific input (e.g. heating) [11,12]. The shape-changing behavior is reversible and programmable by control of liquid crystal (LC) alignment during the synthetic procedure [13,14,15]. It is then clear that molecular alignment is a key parameter to manipulate both the 2D-to-3D folding and the adaptive shaping in response to stimuli. For this purpose, the most employed synthetic strategy is based on the polymerization of acrylate mesogens, [16] which results in materials with large elastic modulus and glassy behavior [17], whose internal strain can be modulated during polymerization.

Among the most interesting examples reported in the literature, spiral structures with different handedness have been prepared from flat LCNs with a twisted alignment. Such structures were demonstrated to be able to wind/unwind under irradiation, thus transforming light energy into a macroscopic helical motion [18]. Exploiting the same alignment, Ware et al. described how to create stretchable electronic devices integrating gold traces on a helical LC polymer that exhibit small variations of resistance during substrate stretching. The same structures can be also used as antennas with different operating frequencies depending on the temperature [19]. A mechanical application of such photo-responsive materials has been described by Primagii et al., employing a radial molecular alignment to realize a flower-like shaped LCN. The actuator autonomously opens and closes depending on the incident light intensity, thus mimicking the eye’s iris [8].

Even if many examples of internal strain engineering have been described and modeled playing on LC alignment [20], less attention has been devoted to the effect of molecular composition on material deformation. In this article, we report a study on the spontaneous curving of flat LCNs having different chemical compositions and their evolution under external stimuli (heat and light). We demonstrate how by using a molecular engineering approach, i.e. modifying the molecular structure of the liquid crystalline monomers (changing the flexible spacer or the ester orientation in the central core), it is possible to modify the strain in the material and thus its natural curving. Previous studies have demonstrated how modification of the mesogen alkil chains can affect the temperature response of the final material [21]. As an example, Keller et al. showed how increasing the number of carbon atoms in the spacers decreases the contraction percentage under heating, with a negligible effect on the transition temperatures for side-on liquid crystalline elastomers (LCEs) [22]. Applying the same molecular changes to materials prepared by diacrylate mesogens leads instead to an increased temperature sensitivity [23]. On the other hand, the effect of the ester bond orientation in acrylate-based LC polymers has been evaluated only in terms of their optical and dielectric properties [24]. In this article, these molecular parameters have been analyzed both in term of mesomorphic and mechanical properties and the material response to stimuli. The results represent a new insight for the development of functional photo-responsive actuators by a 2D-to-3D shape shifting approach.

## 2. Materials and Methods

**Figure 1:**
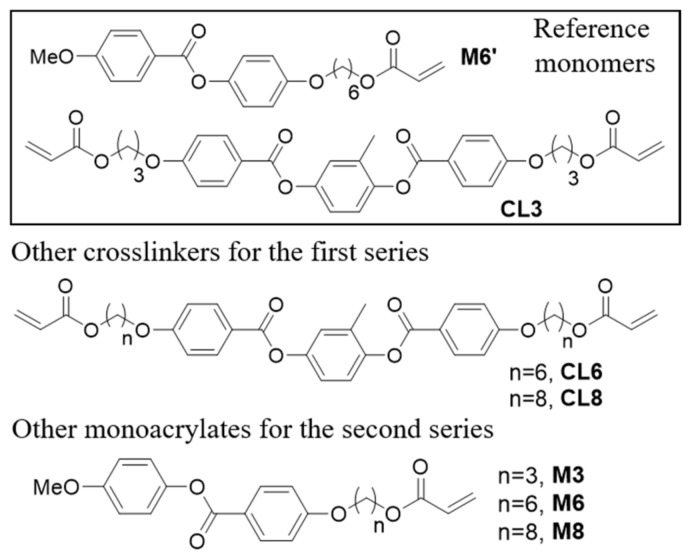
Molecular structure of monomers employed.

Materials and general procedure: Molecular structures of the monomers are represented in Figure 1 and Figure S1. Mesogenic monoacrylates are called Mx, where x represents the number of carbon atoms in the alkyl spacer. Compound M6′ is reported with ‘ to distinguish the different ester orientations (in the aromatic core) with respect to the other monoacrylates. Analogously, crosslinkers are noted as Cly, where y represents the number of carbon atoms in the alkyl spacer. Polymeric materials are called LCNx(‘)-y, where x and y are the numbers of carbons in the spacers, respectively, of the monoacrylate and the crosslinker (‘ is used to distinguish between materials containing M6 or M6′).

Monomers M6′, CL3, and CL6 were purchased by Synthon Chemical (Bitterfeld-Wolfen, Germany), Irgacure 369 was purchased from Sigma Aldrich (Tokyo, Japan), and the azo dye was prepared as previously described [25]. Synthesis and mesomorphic properties of the other mesogens are reported in Supporting Information (Figure S2). Thermal transitions were measured using a DSC TA Instruments Q-2000 calorimeter (TA Instruments, Milano, Italy) under a nitrogen atmosphere (heating and cooling rate: 10 °C/min). Polarized optical microscopy (POM) was performed using an inverted microscope (Zeiss, Axio Observer A1) with cross polarizers equipped with a Linkam PE120 hot stage (Linkam Scientific, Tadworth, UK). Dynamic mechanical thermal analysis (DMTA) was performed using a Rheometric DMTA V analyzer (Rheometric Scientific, UK). The measurements were carried out in tensile mode using rectangular tension geometry. Specimens with size of 20 × 5 × 0.02 mm^3^ were employed. A strain amplitude of 0.1% (linear viscoelastic range), a strain frequency of 1 Hz, and a scanning rate of 4 °C/min were chosen for all measurements. A static force of 20 g was applied. The dynamic mechanical behavior was analyzed in a temperature range between −50 and 50 °C.

LCN film preparation: The scheme for material preparation is reported in Figure S3. Liquid crystalline cells were prepared by two glasses (with different coating) and 20 μm glass spheres as spacers. The coating was chosen in order to reach a specific alignment. For homogeneous cells, two glasses coated by polyvinyl alcohol (PVA) and rubbed with a velvet cloth were employed, while splayed cells were prepared by a rubbed PVA-coated glass on the bottom and a polyimide (PI1211, Nissan Chemicals)-coated glass on the top. The monomer mixtures were melted on a hot plate at 70 °C and then infiltrated for capillarity in the cells. Afterwards, the samples were cooled down until reaching the desired alignment and polymerized for 10 min with an UV LED lamp. All mixtures were irradiated at 45 °C, with an exception for LCN3-3, which was irradiated at room temperature. The cells were then heated at 65 °C, irradiated for a further 10 min and then opened to detach the final polymeric film.

Thermal and light-induced deformations: Millimetric cantilevers were prepared by cutting splayed films with longer dimension along the director of the planar homogenous side. Thermal deformation was observed directly on a hot stage, while light response was tested by illumination with a DPSS 532 nm laser from the top, varying the intensity with a neutral density wheel-filter.

## 3. Results and Discussion

### 3.1. Material Preparation

Materials under study were prepared by photopolymerization of LC mixtures after their alignment in LC cells. The employed molecules, shown in Figure 1, were functionalized with acrylate groups to create the polymeric network by photopolymerization. We focused our investigation starting from a reference mixture widely employed in previous studies, [25,26,27] and containing M6′ (78% mol/mol), CL3 (20% mol/mol), an azobenzene dye (1% mol/mol) as a photo-responsive molecule, and Irgacure 369 (1% mol/mol) as a photoinitiator. Effects of the variation of the monomer ratio have already been investigated in both lithographic micro-patterning [26] and macroscopic film preparation [27], demonstrating how this parameter highly affects both the mechanical properties and the response times to light irradiation. Herein, we focus the attention only on molecular changes of mesogen, keeping fixed the percentage composition with respect to the reference material. Two different series of samples were investigated. In the first one, we substituted the crosslinker (CL3 was replaced with CL6 and CL8) to study the effect of the spacer on the diacrylate compounds. In the other, keeping CL3 fixed, we monitored the effect of the alkyl spacer on monomers (using M3, M6, and M8). Comparing samples from the two series, the effect of the ester orientation can also be extrapulated.

**Figure 2:**
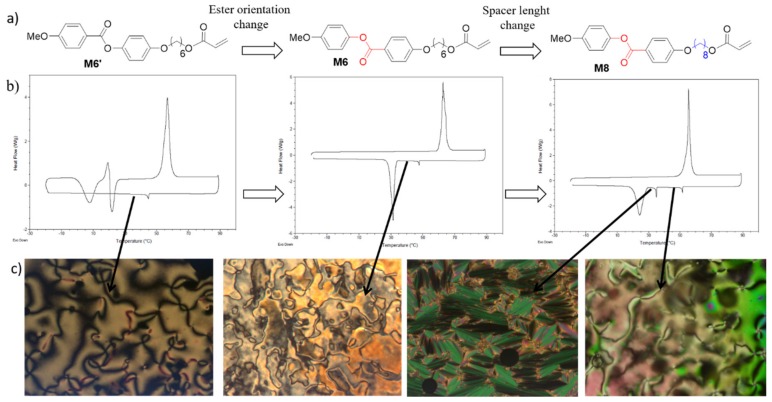
Examples of structure-mesomorphic properties relationship in low-molecular weight liquid crystales. (**a**) Molecular structures of three mesogenic acrylates. (**b**) Differential scanning calorimetry (DSC) traces related to the second heating and cooling scan (10 °C/min) of M6′, M6, and M8 (from left to right). (**c**) Representative polarized optical microscopy (POM) images of the LC phases: nematic texture in M6′, nematic texture in M6, smectic A, and nematic textures in M8 (from left to right).

A representative example of the importance of the analyzed molecular parameters is show in Figure 2, which compares the liquid crystalline properties of some monoacrylates. Mesophases were identified by differential scanning calorimetry (DSC) and polarized optical microscopy (POM) analysis, which is reported in Figure 2b,c.

The first molecule, M6′, is a commercial compound widely explored for LCN preparation, and it is characterized by a monotropic nematic phase below 44 °C. At room temperature, the compound is still in the LC phase and crystallization occurs only after some hours. For compound M6, in which only the orientation of the ester in the aromatic core has changed, crystallization on cooling is more favored and th LC phase is mantained only up to 33 °C. M8 has the same ester orientation of M6, but a longer alkyl spacer. As in the previous case, the ester orientation affects the crystallization process (leading to a crystal compound at 28 °C on cooling), while the extended aliphatic spacer induces a polymorphism in the LC phases. Both nematic and a smectic A phases are observed on cooling. The same molecular changes affect also different behaviors of liquid crystalline polymers, modifing their mesomorphic and dieletric properties [28,29].

LCNs with both homogeneous planar and splayed alignment were prepared. The mixtures were characterized by DSC analysis (see Table S1 and Figure S4) to select the appropriate processing temperature range. All mixtures exhibited only one LC phase (a nematic one), and the inversion of the ester orientation led to crystallization at room temperature for the mixtures of the second series. Even if this fact does not compromise our studies on macroscopic samples, we have to notice that micro-nano structuring of LCNs with standard litographic tecniques is facilitated by a room temperature nematic phase [25,26]. The control of the ester orientation in the mesogenic core thus enables the easy expansion of the study on the microscopic scale. To prepare macroscopic films, the mixtures were melted in the isotropic phase and infiltrated into liquid crystalline cells prepared with different coating layers according to the desired alignment. Then, the samples were cooled down until the formation of a monodomain texture, and polymerized by irradiation with a UV LED lamp. A scheme of the fabrication process and representative POM images of a LCN film are reported in Figure S3. The polymeric films were removed from the cell and used for different mechanical characterizations without a further purification process. Polymerization temperature is very important to determine if the film self-folding, and it was set at 45 °C for all samples, except for LCN3-3, which was polymerized at room temperature due to the lower transition temperature of its monomeric mixture.

### 3.2. Thermo-Mechanical Analysis of the Polymers

Dynamic -mechanical thermal analysis (DMTA) and DSC were used to evaluate the thermo-mechanical properties of samples as a function of the molecular parameters. The measurements were carried out in the parallel direction with respect to the liquid crystalline alignment as a function of temperature in tensile mode. The glass transition in DMTA experiments was taken as the maximum of tan δ peak, whereas in the DSC measurements the midpoint of the transition was taken.

**Figure 3:**
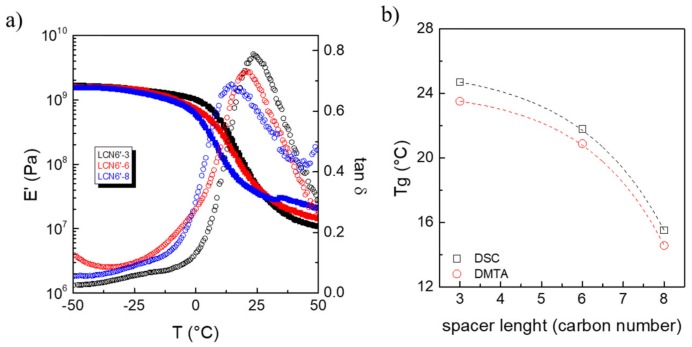
Thermo-mechanical properties of the polymer with different crosslinkers in the parallel direction with respect to the liquid crystalline alignment. (**a**) Dynamic storage modulus E’ and loss tangent tan *δ* as a function of temperature for LCN6′-3 (black), LCN6′-6 (red), and LCN6′-8 (blue) samples (E’ full square symbols and tan *δ* open circle symbols). (**b**) Glass transition temperature as a function of spacer length in the crosslinker agent measured by DSC (open black square) and DMTA (open red circle).

The dynamic storage modulus E’ and loss tangent tan δ as a function of temperature for samples with different spacer length in the crosslinker are reported in Figure 3a. Figure 3b reports the trends of the glass transition by DSC and DMTA as a function of the alkyl spacer length in the crosslinker.

As exepected, the dynamic storage modulus E’ decreases as a function of temperature and a drop of two orders of magnitude is observed in the temperature range between 0 and 50 °C due to the glass transition. In the glassy state (T = −50 °C), E’ decreases regularly from 1.7 to 1.5 GPa as the carbon number in the crosslinker increases [30], whereas a parallel modulus increase from 10 to 20 MPa (T = +50 °C) is observed in the rubbery region. The glass transition temperature decreases as the spacer length increases in the crosslinker [30] from 25 to 15 °C, in agreement with the DSC analysis (Figure 3B and Figure S5). A *β* transition that partially overlaps to the glass transition (*α* transition) is recorded for all samples, due to relaxation of the mesogenic units. This transition partially overlaps to the glass transition (*α* transition) [31,32].

**Figure 4:**
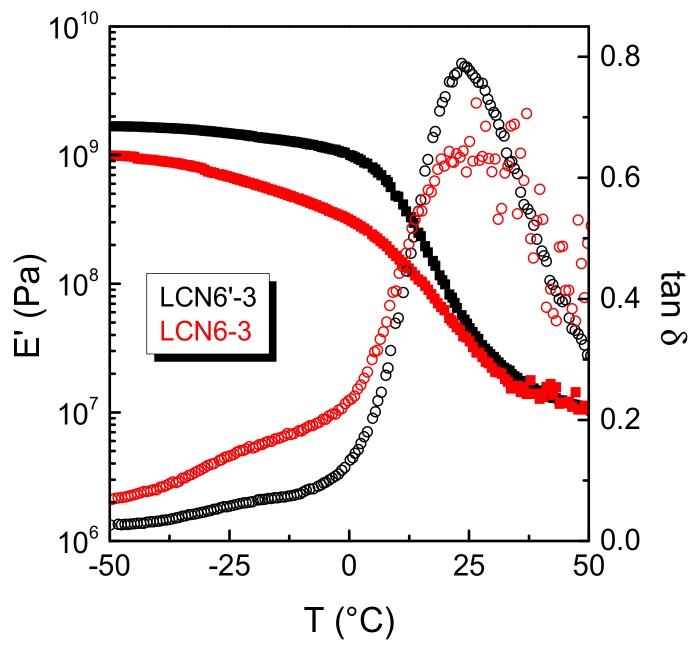
Dynamic storage modulus E’ and loss tangent tan δ in the parallel direction with respect to the liquid crystalline alignment as a function of temperature for LCN6′-3 (black) and LCN6-3 (red) samples (E’ fully square symbols and tan δ open circle symbols).

Figure 4 illustrates the trend of E’ and tan *δ* for LCN6′-3 and LCN6-3 samples obtained using the same crosslinker CL3, but differing in the monomer, in order to evaluate the effect of orientation of the ester group linking the two aromatic rings. The ester orientation in the monomer plays an important role on the mechanical properties of the material. Before the glass transition, E’ for LCN6′-3 sample is two times larger than the one of the sample prepared from monomer M6, while the glass transition is not affected by the ester orientation.

**Figure 5:**
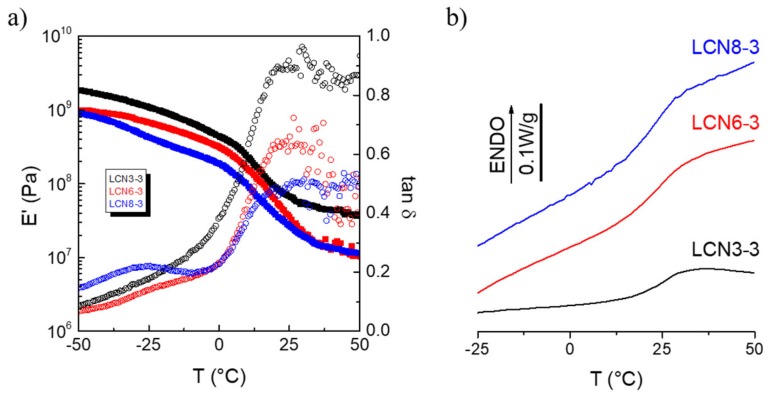
Thermo-mechanical properties of the polymers prepared with different monoacrylates. (**a**) Dynamic storage modulus E’ and loss tangent tan δ in the parallel direction with respect to the liquid crystalline alignment as a function of temperature for LCN3-3 (black), LCN6-3 (red), and LCN8-3 (blue) samples (E’ fully square symbols and tan δ open circle symbols). (**b**) DSC second heating curves for LCN3-3 (black line), LCN6-3 (red line), and LCN8-3 (blue line).

The analysis of E’ and tan *δ* as functions of temperature for samples with different spacer lengths in the monoacrylate monomer is reported in Figure 5, together with the DSC second heating curves (Figure 5b).

The trends of E’ and tan *δ* as function of the monoacrylate present slightly different behaviors than those observed for the crosslinker change. In the glassy state (T = −50 °C), E’ decreases from 1.8 to 0.9 GPa as the carbon number in the monoacrylate increases, and the same trend was observed for the modulus in the rubbery region (that decrease from 37 to 10 MPa at T = +50 °C). A slight decrease of the glass transition temperature is recorded as a function of carbon number (from 24 to 22 °C), demostranting that the modification of the crosslinker structure is a more efficent way to modulate the glass transition.

### 3.3. Self-Curving of LCN Films and Shape-Change in Response to Heating

**Figure 6:**
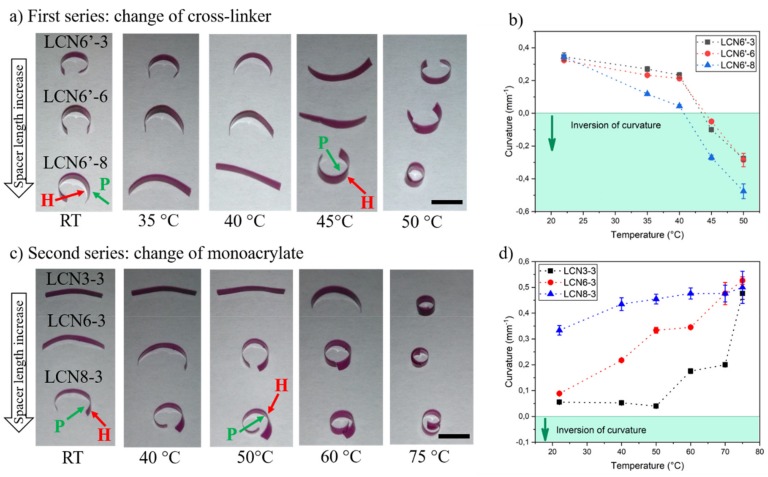
Curving behavior of splayed polymers and their shape-change under heating. (**a**) Optical images of cantilevers made by LCN6′-3, LCN6′-6, and LCN6′-8 (from top to the bottom) on a hot stage at different temperatures. Scale bar: 4 mm. (**b**) Graph of curvature radius for splayed LCNs of series 1 at different temperatures. (**c**) Optical images of cantilevers made by LCN3-3, LCN6-3, and LCN8-3 (from top to the bottom) on a hotstage at different temperatures. Scale bar: 5.2 mm (**d**) Graph of curvature radius for splayed LCNs of series 2 at different temperatures. The radii were calculated by optical images drawing the circumference inscribed into the cantilevers. Green and red arrows indicate, respectively, the planar homogeneous (P) and hometropic (H) face of the cantilever.

Internal strain of splayed LCNs can be released in a natural curved conformation by heating the film above the glass transition temperature. For example, the formation of gradient strain in splayed films prepared by diacrylate mesogens can be explained by a larger polymerization shrinkage along the molecular orientation than in their perperdicular directions [33]. The splayed alignment was chosen as the most efficent one for determining bending movement as a function of temperature [23]. For all formulations, the longest dimension of the stripes was parallel to the director of the homogenous planar side and, after heating at 50 °C, the strain was released to give curved cantilevers. Figure 6a,c show the optical images of all materials at different temperatures, while Figure 6b,d report the values of the curvature extrapolated by the images and defined as the inverse of the radius of the circumference inscribed into the cantilever. Negative values of the curvature are reported in case of inversion of the curvature direction with respect to the starting configuration at room temperature.

In the first series, we observed that increasing the flexible spacer does not create substantial differences in the curving behavior at room temperature. In all cases, films spontaneouly bend towards the homeotropic (H) side as previously observed in many studies [8]. When increasing the temperature, the homogenous planar (P) film side tends to contract, while the other face, the homeotropic one, tends to expand and as a result, the film bends toward the homogenoues side. Within this series, the film first becomes flat and then curved in the opposite direction. Increasing the spacer length enhances the temperature sensitivity within the LCN6′-8 stripe that started to deform at 35 °C and is almost flat at 40 °C, while within the same range the LCN6′-6 and LCN6′-3 stripes are still bent toward the homeotropic side. For the three formulations, inversion of curvature is completed at 50 °C. Very interestingly, the stripe shape-change also occurs at physiological temperature and could be exploited for LCN applications in biology, from static cell scaffolds [34,35] to dynamic platforms.

A different behavior was found in the case of the second series. Therein, the different formulations give rise to different self-folding. For LCN3-3, polymerized at room temperature, the cantilever presents an almost flat shape, and while increasing the spacer length, the initial curvature increases. The most surprising effect is the initial curvature towards the homogeneous planar side rather than the hometropic one, as in the first series. This peculiarity is observed both for LCN6-3 and LCN8-3, highlighting that the orientation of the ester moiety can determine the natural curving direction (after strain release). The behavior could be explained as an opposite polymerization shrinkage along the molecular director, with respect to findings reported by other groups for splayed LCNs [33] and also observed in series 1. On the other hand, the curvature change under heating follows the classical trend of the other materials. As expected, as the temperature increases, the stripe bends towards the homogeous planar side. Therefore, the cantilever cannot flatten and invert its intial bending, but continues to curve up to the structure rolling. Also in this series, the spacer influences the temperature response and the LCN3-3 stripe is still almost flat at 50 °C, while the other samples have already started their bending. Around 80 °C, the 3 samples rolled in on themselves. 

**Figure 7:**
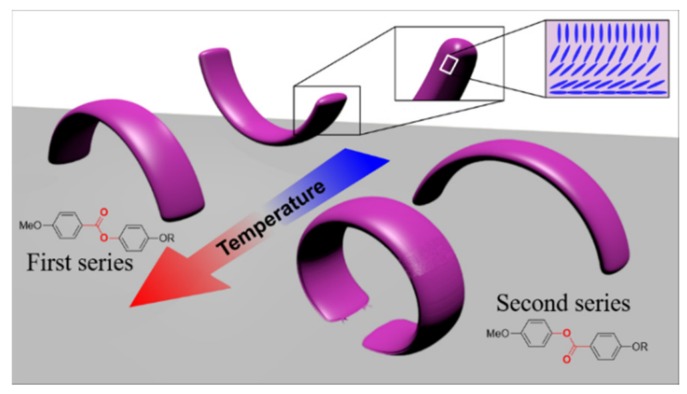
Scheme of the possible curving behavior depending on the ester orientation into the mesogenic core.

Figure 7 summarizes the ester orientation effect in the two series. The initial curvature is determined by the chemical strain of the network that creates different results depending on the ester orientation, while in both cases the bending under heating happens towards the homogenous planar side.

### 3.4. Shape-Change of LCN Films in Response to Light Irradiation

Azobenezene moieties into material formulations introduce an optical response of LCN stripes [36,37]. In this specific case, the dye has a strong absorption in the visible range, and therefore a green light was employed to activate the shape-change [25]. Both laser and incoherent light irradiation (by an LED lamp) can be used to drive the deformation [38]. Within the selected formulations, the mechanism of the material reshaping has to be ascribed to thermal heating (where light energy is dissipated inside the network as heat) [39], rather than an optical effect, due to the trans-to-cis isomerization that, on the other hand, affords a photo-softening effect at low light power [27]. As the laser power increase, the material temperature gradually increases, disordering the molecular arrangement and thus leading to different curvature radii [27]. The stripe response time is in the range of seconds and, after switching off the laser, the complete recovery of the initial shape is observed (without further light irradiation) [27].

The combination of materials with differentiated mechanical responses opens itself up to complex photonic robots and self-regulating devices (where the actuator itself operates depending of the environmental stimulus dose) [40]. Considering a flat external condition, (which is easier to produce and more common in nature), the asymmetric operation of the robot should be encoded in the intrinsic properties of the materials. If different parts of the robot deform differently under the same flat stimulus, a complex deformation can be attained using a single external source. This characteristic result is fundamental at the microscale, where local control of the environmental conditions is hard to achieve and the application of a flat stimulus is highly demanded. On the other side, independent control of the deformation of a composite structure can be achieved by doping LCNs with dyes with complementary absorption and employing multiple wavelength irradiation [41].

**Figure 8:**
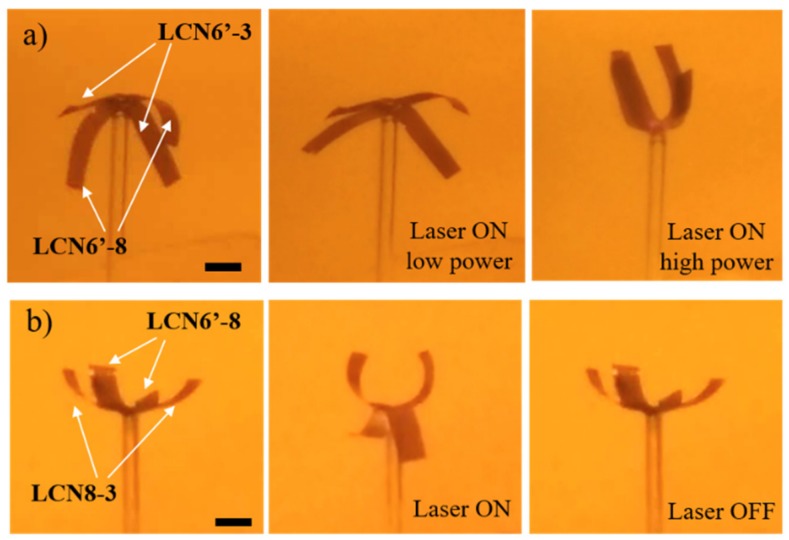
Light response of liquid crystalline networks (LCNs). (**a**) Images of two LCN stripes (LCN6′-3 and LCN6′-8) simultaneously irradiated at different laser powers (0.53 and 1.58 mWmm^−2^). The two materials afforded different bending angles at the same power. Scale bars: 2.7 mm. (**b**) Images of two LCN stripes (LCN6′-8 and LCN8-3′) simultaneously irradiated. The two materials bend in opposite directions at the same power and recover their original position after switching off the laser. Scale bars: 2.7 mm.

A simpler option is provided by materials with different dose-dependent responses to monochromatic light. In this case, a diverse deformation is induced by the light intensity in different part of the same structure, as reported in Figure 8a and Video S1. As a demonstration, two curved stripes of LCN6′-3 and LCN6-8′ (both from the first series) are arranged in a cross-like structure that was irradiated completely from the top with green light. Low irradiation power (0.53 mWmm^−2^) generates a bending angle of the LCN6′-8 stripes around 50°, while the other ones are subjected only to small deformation, demonstrating that using materials with different photo-mechanical responses could be a valuable way toward independent actuation using the same color. The same actuation can be obtained in both materials applying a laser intensity (1.58 mWmm^−2^) that saturates both deformations. Even if modulation of the light-dose response can also be achieved by other strategies (such as by variation of the dye content [42]), the proposed approach focuses on the improvement of shape-deformation at lower temperatures, which is required for the use of reduced energy in photothermal effects. An additional degree of freedom should be introduced by means of dichroic dyes controlled by polarized light [39].

Another successful method to induce asymmetric motion is to program different molecular orientations in selected parts of the actuator by photoalignment technique [43]. However, photoalignment requires several steps and delicate procedures. Thus, we demonstrated that an asymmetric deformation can be obtained under a flat light stimulus using an uniform splayed alignment by combining materials with different molecular compositions. As a proof of concept, a flower-like structure has been made by two stripes of LCN6′-3 (from the first series) and LCN8-3 (from the second series). Figure 8b and Video S2 show that the different stripes were assembled to have curvature towards the same direction and were then irradiated from the top with a green laser (power: 1.58 mWmm^−2^). The petal bending evolved in opposite directions. Two of them (LCN8-3) reduced their radius of curvature, while LCN6′-8 stripes reversed their curvature. In this case, the deformation directions are determined only by the molecular composition, and they are not affected from the irradiation side, as illustrated in Figure S6, where the same structure is irradiated from the opposite side. Once the light is switched off, all petals recover their initial position.

Both examples demonstrate how combining parts with different compositions is a straightforward strategy to engineer actuators able to perform asymmetric deformations. These concepts could be exploited for the realization of non-reciprocal motion under a flat stimulus, a fundamental requirement towards the realization of miniaturized robotic swimmers [44,45].

## 4. Conclusions

In this paper, the mechanical properties of LCNs with different compositions were studied, as well as their self-curving behavior. Among the analyzed molecular parameters, both the length of the mesogenic flexible chain and the orientation of the ester in the aromatic core affect the material’s shape-changing behavior. On one side, the spacer-length increase in the crosslinker can be exploited to enhance the stimuli response of the material (by both heating and light irradiation), while the ester orientation modifies the spontaneous self-curving towards the planar homogeneous or the homeotropic side. Under light irradiation, the actuators increase their curvature or become flat and then change their bending direction. The possibility to design a selected self-folding LCN only by changing the molecular composition of the starting monomeric mixture could be combined with different LCN manufacturing technologies, from fiber drawing to 3D printing, to work towards the realization of new actuators. The combination of different materials will allow for the creation of complex deformations, opening up non-symmetrical motion under a monochromatic light flat stimulus.

## Supplementary Materials

The following are available online at https://www.mdpi.com/2073-4360/11/10/1644/s1. Figure S1. Structure of the monomeric components of the mixtures. Figure S2. Synthesis of liquid crystalline monomers. Table S1. Composition and thermal properties of monomer mixtures. Figure S3. Further details on LCN preparation. Figure S4. DSC traces for the monomeric mixtures. Figure S5. DSC second heating curves for LCN6′-3, LCN6′-6 and LCN6′-8. Figure S6. Light response of two LCN stripes (LCN6′-8 and LCN8-3′) simultaneously irradiated from the top with a green laser. Video S1. Light response of two LCN stripes (LCN6′-3 and LCN6′-8) simultaneously irradiated with a green laser at different laser powers. Video S2. Light response of two LCN stripes (LCN6′-8 and LCN8-3′) simultaneously irradiated with a green laser.

## References

[1] Lee J.-Y., An J., Chua C.K. (2017). Fundamental and applications of 3D printing for novel materials. Appl. Mater. Today.

[2] Barner-Kowollik C., Bastmeyer M., Blasco E., Delaittre G., Müller P., Richter B., Wegener M. (2017). 3D laser micro-and nanoprinting: Challenges for chemistry. Angew. Chem. Int. Ed..

[3] Cheng X., Zhang Y. (2019). Micro/Nanoscale 3D Assembly by Rolling, Folding, Curving, and Buckling Approaches. Adv. Mater..

[4] Yuan B., Jin Y., Sun Y., Wang D., Sun J., Wang Z., Zhang W., Jiang X. (2012). A strategy for depositing different types of cells in three dimensions to mimic tubular structures in tissues. Adv. Mater..

[5] Ko H.C., Stoykovich M.P., Song J., Malyarchuk V., Choi W.M., Yu C.J., Geddes J.B., Xiao J., Wang S., Huang Y. (2008). A hemispherical electronic eye camera based on compressible silicon optoelectronics. Nature.

[6] Jamal M., Zarafshar A.M., Gracias D.H. (2011). Differentially photo-crosslinked polymers enable self-assembling microfluidics. Nat. Commun..

[7] Hippler M., Blasco E., Qu J., Tanaka M., Barner-Kowollik C., Wegener M., Bastmeyer M. (2019). Controlling the shape of 3D microstructures by temperature and light. Nat. Commun..

[8] Zeng H., Wani O.M., Wasylczyk P., Kaczmarek R., Priimagi A. (2017). Self-regulating iris based on light-actuated liquid crystal elastomer. Adv. Mater..

[9] Anglaret E., Brunet M., Desbat B., Keller P., Buffeteau T. (2005). Molecular orientation in liquid crystal elastomers. Macromolecules.

[10] Küpfer J., Finkelmann H. (1991). Nematic liquid single crystal elastomers. Die Makromol. Chemie Rapid Commun..

[11] Ohm C., Brehmer M., Zentel R. (2010). Liquid crystalline elastomers as actuators and sensors. Adv. Mater..

[12] White T.J., Broer D.J. (2015). Programmable and adaptive mechanics with liquid crystal polymer networks and elastomers. Nat. Mater..

[13] Ware T.H., McConney M.E., Wie J.J., Tondiglia V.P., White T.J. (2015). Voxelated liquid crystal elastomers. Science.

[14] Mostajeran C., Warner M., Modes C.D. (2017). Frame, metric and geodesic evolution in shape-changing nematic shells. Soft Matter.

[15] Nocentini S., Martella D., Wiersma D.S., Parmeggiani C. (2017). Beam steering by liquid crystal elastomer fibres. Soft Matter.

[16] Liu D., Broer D.J. (2014). Liquid crystal polymer networks: Preparation, properties, and applications of films with patterned molecular alignment. Langmuir.

[17] Hikmet R.A.M., Broer D.J. (1991). Dynamic mechanical properties of anisotropic networks formed by liquid crystalline acrylates. Polymer.

[18] Iamsaard S., Aßhoff S.J., Matt B., Kudernac T., Cornelissen J.J., Fletcher S.P., Katsonis N. (2014). Conversion of light into macroscopic helical motion. Nat. Chem..

[19] Kim H., Gibson J., Maeng J., Saed M., Pimentel K., Rihani R., Pancrazio J.J., Georgakopoulos S., Ware T.H. (2019). Responsive, 3D Electronics Enabled by Liquid Crystal Elastomer Substrates. ACS Appl. Mater. Interfaces.

[20] Sawa Y., Ye F., Urayama K., Takigawa T., Gimenez-Pinto V., Selinger R.L., Selinger J.V. (2011). Shape selection of twist-nematic-elastomer ribbons. Proc. Natl. Acad. Sci. USA.

[21] Broer D.J., Mol G.N. (1991). In-situ photopolymerization of oriented liquid-crystalline acrylates, 5. Influence of the alkylene spacer on the properties of the mesogenic monomers and the formation and properties of oriented polymer networks. Makromol. Chem..

[22] Renbo W., Zhou L., He Y., Wang X., Keller P. (2013). Effect of molecular parameters on thermomechanical behavior of side-on nematic liquid crystal elastomers. Polymer.

[23] Mol G.N., Harris K.D., Bastiaansen C.W., Broer D.J. (2005). Thermo-mechanical responses of liquid-crystal networks with a splayed molecular organization. Adv. Funct. Mater..

[24] Hikmet R.A.M., Lub J., Tol A.J.W. (1995). Effect of the orientation of the ester bonds on the properties of three isomeric liquid crystal diacrylates before and after polymerization. Macromolecules.

[25] Zeng H., Martella D., Wasylczyk P., Cerretti G., Lavocat J.C.G., Ho C.H., Parmeggiani C., Wiersma D.S. (2014). High-resolution 3D direct laser writing for liquid-crystalline elastomer microstructures. Adv. Mater..

[26] Nocentini S., Martella D., Parmeggiani C., Wiersma D. (2016). Photoresist design for elastomeric light tunable photonic devices. Materials.

[27] Martella D., Antonioli D., Nocentini S., Wiersma D.S., Galli G., Laus M., Parmeggiani C. (2017). Light activated non-reciprocal motion in liquid crystalline networks by designed microactuator architecture. RSC Adv..

[28] Lam J.W., Kong X., Dong Y., Cheuk K.K., Xu K., Tang B.Z. (2000). Synthesis and properties of liquid crystalline polyacetylenes with different spacer lengths and bridge orientations. Macromolecules.

[29] Braun L., Linder T., Hessberger T., Zentel R. (2016). Influence of a crosslinker containing an Azo group on the actuation properties of a photoactuating LCE system. Polymers.

[30] Skandani A., Clement J.A., Tristam-Nagie S., Shankar M.R. (2017). Aliphatic flexible spacer length controls photomechanical response in compact, ordered liquid crystalline polymer networks. Polymer.

[31] Harris K.D., Cuypers R., Scheibe P., Van Oosten C.L., Bastiaansen C.W.M., Lub J., Broer D.J. (2005). Large amplitude light-induced motion in high elastic modulus polymer actuators. J. Mater. Chem..

[32] Van Oosten C.L., Harris K.D., Bastiaansen C.W.M., Broer D.J. (2007). Glassy photomechanical liquid-crystal network actuators for microscale devices. Eur. Phys. J..

[33] Hikmet R.A.M., Zwerver B.H., Broer D.J. (1992). Anisotropic polymerization shrinkage behaviour of liquid-crystalline diacrylates. Polymer.

[34] Martella D., Paoli P., Pioner J.M., Sacconi L., Coppini R., Santini L., Lulli M., Cerbai E., Wiersma D.S., Poggesi C. (2017). Liquid crystalline networks toward regenerative medicine and tissue repair. Small.

[35] Martella D., Pattelli L., Matassini C., Ridi F., Bonini M., Paoli P., Baglioni P., Wiersma D.S., Parmeggiani C. (2019). Liquid Crystal-Induced Myoblast Alignment. Adv. Health. Mater..

[36] Ikeda T., Mamiya J.I., Yu Y. (2007). Photomechanics of liquid-crystalline elastomers and other polymers. Angew. Chem. Int. Ed..

[37] Yu H., Ikeda T. (2011). Photocontrollable liquid-crystalline actuators. Adv. Mater..

[38] Ferrantini C., Pioner J.M., Martella D., Coppini R., Piroddi N., Paoli P., Calamai M., Pavone F.S., Wiersma D.S., Tesi C. (2019). Development of Light-Responsive Liquid Crystalline Elastomers to Assist Cardiac Contraction. Cir. Res..

[39] Martella D., Nocentini S., Micheletti F., Wiersma D.S., Parmeggiani C. (2019). Polarization-dependent deformation in light responsive polymers doped by dichroic dyes. Soft Matter.

[40] Martella D., Nocentini S., Parmeggiani C., Wiersma D.S. (2019). Self-Regulating Capabilities in Photonic Robotics. Adv. Mater. Technol..

[41] Gelebart A.H., Mulder D.J., Vantomme G., Schenning A.P., Broer D.J. (2017). A Rewritable, Reprogrammable, Dual Light-Responsive Polymer Actuator. Angew. Chem. Int. Ed..

[42] Kondo M., Sugimoto M., Yamada M., Naka Y., Mamiya J.I., Kinoshita M., Shishido A., Yu Y., Ikeda T. (2010). Effect of concentration of photoactive chromophores on photomechanical properties of crosslinked azobenzene liquid-crystalline polymers. J. Mater. Chem..

[43] Wani O.M., Zeng H., Wasylczyk P., Priimagi A. (2018). Programming photoresponse in liquid crystal polymer actuators with laser projector. Adv. Opt. Mater..

[44] Nocentini S., Parmeggiani C., Martella D., Wiersma D.S. (2018). Optically driven soft micro robotics. Adv. Opt. Mater..

[45] Palagi S., Fischer P. (2018). Bioinspired microrobots. Nat. Rev. Mater..

