# Supplementary material for: Opposite Self-Folding Behavior of Polymeric Photoresponsive Actuators Enabled by a Molecular Approach
Source: Polymers (Basel). 2019 Oct 10;11(10):1644. doi: 10.3390/polym11101644 (PMC6835338; doi:10.3390/polym11101644)
Supplement: Supplementary file 1 [file polymers-11-01644-s001.zip › Martella_SI_revised.doc]

**SUPPORTING INFORMATION**

**Opposite self-folding behavior of polymeric photoresponsive actuators enabled by a molecular approach**

**Daniele Martella,* Sara Nocentini, Diego Antonioli, Michele Laus, Diederik S. Wiersma and Camilla Parmeggiani**

**Figure S1.** Structure of other monomeric components of the mixtures.

**SYNTHESIS OF MESOGENIC COMPOUNDS**

**Figure S2.** Synthesis of liquid crystalline monomers. Reagents and conditions: a) chloro-alcohol, KOH, KI, EtOH:H_2_O 5:1, reflux, 18 h, 69% for **1**, 79% for **2**, 74% for **3**; b) acryloyl chloride, NMP, rt, 3 h, 100% for **4**, **5** and **6**; c) methoxyphenol, DCC, 4-pyrrolidinopyridine, CH_2_Cl_2_, rt, 18 h, 82% for **M3**, 79% for **M6**, 63% for **M8**; d) methylhydroquinone, DCC, 4-pyrrolidinopyridine, CH_2_Cl_2_, rt, 18 h, 77%.

**4-(3-Hydroxypropoxy)benzoic acid (1)**. 4-Hydroxybenzoic acid (5 g, 36.1 mmol) was dissolved in a mixture of EtOH (30mL) and water (6 mL). KOH (5.3 g, 94.1 mmol), KI (catalytic amount) and 3-chloro-1-propanol (5.1 g, 54.3 mmol) were added and the solution was heated at reflux for 18 h. Then, EtOH was removed under reduced pressure and the resulting mixture was poured into water and extracted with Et_2_O. The water-phase solution was acidified with diluted HCl to give a white precipitate, which was filtrated and washed with water to afford the product in 69% yield (4.9 g, 24.9 mmol). ^1^H-NMR (300 MHz, CD_3_OD) δ 7.96-7.94 (m, 2H, Ar), 6.99-6.97 (m 2H, Ar), 4.14 (t, *J* = 6.2 Hz, 2H, ArOC*H_2_*A), 3.74 (t, *J* = 6.2 Hz, 2H, AC*H_2_*OH), 2.00 (quint, *J* = 6.2 Hz, 2H, aliphatic –C*H_2_*-) ppm.

**4-[(6-Hydroxyhexyl)oxy]benzoic acid (2).** It was synthesized by the same procedure described for **1** using 1-chloro-6-hexanol instead of 3-chloro-1-propanol. Yield: 79%; ^1^H-NMR (300 MHz, CD_3_OD) δ 7.98-7.93 (m, 2H, Ar), 6.98-6.94 (m 2H, Ar), 4.05 (t, *J* = 6.4 Hz, 2H, ArOC*H_2_*A), 3.56 (t, *J* = 6.5 Hz, 2H, AC*H_2_*OH), 1.88–1.75 (m, 2H, aliphatic –C*H_2_*-), 1.61-1.43 (m, 6H, aliphatic–C*H_2_*-) ppm.

**4-[(8-Hydroxyoctyl)oxy]benzoic acid (3).** It was synthesized by the same procedure described for **1** using 1-chloro-8-octanol instead of 3-chloro-1-propanol. Yield: 74%; ^1^H-NMR (300 MHz, CD_3_OD) δ 7.96-7.94 (m, 2H, Ar), 6.97-6.94 (m 2H, Ar), 4.04 (t, *J* = 6.4 Hz, 2H, ArOC*H_2_*A), 3.54 (t, *J* = 6.6 Hz, 2H, AC*H_2_*OH), 1.83-1.76 (m, 2H, aliphatic –C*H_2_*-), 1.53-1.37 (m, 10H, aliphatic –C*H_2_*-) ppm.

**4-[3-(Acryloyloxy)propoxy]benzoic acid (4).** To a solution of **1** (4.1 g, 20.9mmol) in 40 mL of N-Methyl-2-pyrrolidone (NMP), acryloyl chloride (2.27 g, 25.1mmol) was added dropwise and the mixture was stirred at room temperature for 3 h. Then, water was added to give a white precipitate that was filtrated and washed thoroughly with water to afford the pure product in quantitative yield (5.2 g, 20.9 mmol). ^1^H-NMR (400 MHz, CDCl_3_) δ 8.07-8.05 (m, 2H, Ar), 6.95-6.93 (m, 2H, Ar), 6.42 (dd, *J* = 17.4, 1.4 Hz, 1H, CH=C*H_2_*), 6.14 (dd, *J* = 17.4, 10.4 Hz, 1H, C*H*=CH_2_), 5.84 (dd, *J* = 10.4, 1.4 Hz, 1H, CH=C*H_2_*), 4.37 (t, *J* = 6.1 Hz, 2H, ArOC*H_2_*A), 4.14 (t, *J* = 6.1 Hz, 2H, AC*H_2_*OCO), 2.20 (quint, *J* = 6.1 Hz, 2H, aliphatic –C*H_2_*-) ppm.

**4-[6-(Acryloyloxy)hexyloxy]benzoic acid *(*5).** It was synthesized by the same procedure described for **4** starting from **2** instead of **1**. Yield: 100%; ^1^H-NMR (300 MHz, CDCl_3_) δ 8.07-8.04 (m, 2H, Ar), 6.94-6.91 (m, 2H, Ar), 6.40 (dd, *J*= 17.5, 1.5 Hz, 1H, CH=C*H_2_*), 6.12 (dd, *J* = 17.5, 10.4 Hz, 1H, C*H*=CH_2_), 5.82 (dd, *J* = 10.4, 1.5 Hz, 1H, CH=C*H_2_*), 4.18 (t, *J* = 6.7 Hz, 2H, ArOC*H_2_*A), 4.04 (t, *J* = 6.4 Hz, 2H, AC*H_2_*OCO), 1.86-1.79 (m, 2H, aliphatic –C*H_2_*-), 1.75-1.67 (m, 2H, aliphatic –C*H_2_*-), 1.53-1.43 (m, 4H, aliphatic–C*H_2_*-) ppm.

**4-{[8-(Acryloyloxy)octyl]oxy}benzoic acid (6).** It was synthesized by the same procedure described for 4 starting from **3** instead of **1**.. Yield: 100%; ^1^H-NMR (400 MHz, CDCl_3_) δ 8.06-8.04 (m, 2H, Ar), 6.94-6.92 (m, 2H, Ar), 6.39 (dd, *J* = 17.4, 1.5 Hz, 1H, CH=C*H_2_*), 6.12 (dd, *J* = 17.4, 10.4 Hz, 1H, C*H*=CH_2_), 5.82 (dd, *J* = 10.4, 1.5 Hz, 1H, CH=C*H_2_*), 4.16 (t, *J* = 6.5 Hz, 2H, ArOC*H_2_*A), 4.02 (t, *J* = 6.4 Hz, 2H, AC*H_2_*OCO), 1.84-1.77 (m, 2H, aliphatic –C*H_2_*-), 1.69-1.64 (m, 2H, aliphatic –C*H_2_*-), 1.49-1.37 (m, 8H, aliphatic–C*H_2_*-) ppm.

**4-Methoxyphenyl 4-[3-(acryloyloxy)propoxy]benzoate (M3).** A mixture of **4** (976 mg, 3.90 mmol), 4-methoxyphenol (532 mg, 4.29 mmol), N,N'-dicyclohexylcarbodiimide (DCC, 885 mg, 4.29 mmol), and 4-pyrrolidinopyridine (16 mg, 0.43 mmol) in dry CH_2_Cl_2_ (40 mL) was stirred at room temperature under N_2_ atmosphere for 18 h. The N,N-dicyclohexylurea was filtered and the filtrate was washed with water (1000 mL), 5% acetic acid solution (100 mL) and water (100 mL). The combined organic layers were dried over Na_2_SO_4_, filtered and evaporated under reduced pressure. The crude was purified by recrystallization from EtOH affording the pure product in 82% yield (1,13 g, 3.18 mmol) as a white solid. ^1^H-NMR (400 MHz, CDCl3) δ 8.15-8.13 (m, 2H, Ar), 7.13-7.10 (m, 2H, Ar), 6.98-6.92 (m, 4H, Ar), 6.42 (dd, J = 17.3, 1.4 Hz, 1H, CH=CH2), 6.14 (dd, J = 17.3, 10.4 Hz, 1H, CH=CH2), 5.85 (dd, J = 10.4, 1.4 Hz, 1H, CH=CH2), 4.39 (t, J = 6.3 Hz, 2H, ArOCH2A), 4.16 (t, J = 6.2 Hz, 2H, ACH2OCO), 3.82 (s, 3H, Me), 2.21 (quint, J = 6.3 Hz, 2H,aliphatic –CH2-) ppm.

**4-Methoxyphenyl 4-{[6-(acryloyloxy)hexyl]oxy}benzoate (M6).** It was synthesized by the same procedure described for **M3** starting from **5** instead of **4**. Yield: 79%; ^1^H-NMR (300 MHz, CDCl_3_) δ 8.04-8.01 (m, 2H, Ar), 7.04-7.02 (m, 2H, Ar), 6.86-6.80 (m, 4H, Ar), 6.32 (dd, *J* = 17.0, 1.6 Hz, 1H, CH=C*H_2_*), 6.04 (dd, *J*= 17.0, 10.5 Hz, 1H, C*H*=CH_2_), 5.71 (dd, *J* = 10.5, 1.6 Hz, 1H, CH=C*H_2_*), 4.08 (t, *J* = 6.6 Hz, 2H, ArOC*H_2_*A), 3.88 (t, *J* = 6.5 Hz, 2H, AC*H_2_*OCO), 3.66 (s, 3H, Me), 1.75-1.65 (m, 2H, aliphatic –C*H_2_*-), 1.63-1.56 (m, 2H, aliphatic –C*H_2_*-), 1.46-1.31 (m, 4H, aliphatic–C*H_2_*-) ppm.

**4-Methoxyphenyl4-{[8-(acryloyloxy)octyl]oxy}benzoate (M8).** It was synthesized by the same procedure described for **M3** starting from **6** instead of **4**. Yield: 63% yield. ^1^H-NMR (400 MHz, CDCl_3_) δ 8.15-8.12 (m, 2H, Ar), 7.12-7.10 (m, 2H, Ar), 6.97-6.92 (m, 4H, Ar), 6.40 (dd, *J* = 17.3, 1.5 Hz, 1H, CH=C*H_2_*), 6.12 (dd, *J* = 17.3, 10.4 Hz, 1H, C*H*=CH_2_), 5.82 (dd, *J* = 10.4, 1.5 Hz, 1H, CH=C*H_2_*), 4.16 (t, *J* = 6.7 Hz, 2H, ArOC*H_2_*A), 4.04 (t, *J* = 6.5 Hz, 2H, AC*H_2_*OCO), 3.82 (s, 3H, Me), 1.86-1.78 (m, 2H, aliphatic –C*H_2_*-), 1.70-1.65 (m, 2H, aliphatic –C*H_2_*-), 1.50-1.38 (m, 8H, aliphatic –C*H_2_*-) ppm.

**2-Methyl-1,4-phenylene bis(4-{[6-(acryloyloxy)octyl]oxy}benzoate) (CL8).** A mixture **6** (3.44 g, 10.74 mmol), methylhydroquinone (666 mg, 5.37 mmol), DCC (2.43 g, 11.8 mmol), and 4-pyrrolidinopyridine (175 mg, 1,18 mmol) in dry CH_2_Cl_2_ (125 mL) was stirred at room temperature under N_2_ atmosphere for 18 h. The N,N-dicyclohexylurea was filtered and the filtrate was washed with water (150 mL), 5% acetic acid solution (150 mL) and water (150 mL). The combined organic layers were dried over Na_2_SO_4_, filtered and evaporated under reduced pressure. The crude was recrystallized from EtOH affording the pure product in 77% yield (3.02 g, 4.14 mmol) as white solid. ^1^H-NMR (400 MHz, CDCl_3_) δ 8.17-8.12 (m, 4H, Ar), 7.17 (d, J = 8.7 Hz, 1H, Ar), 7.13 (d, J = 2.6 Hz, 1H, Ar), 7.08 (dd, J = 8.7, 2.6Hz, 1H, Ar), 6.99-6.96 (m, 4H, Ar), 6.40 (dd, J = 17.2, 1.5 Hz, 1H, CH=CH2), 6.12 (dd, J = 17.2, 10.4 Hz, 1H, CH=CH2), 5.82 (dd, J = 10.4, 1.5 Hz, 1H, CH=CH2), 4.16 (t, J = 6.7 Hz, 4H, ArOCH_2_A), 4.05 (t, J = 6.5 Hz, 2H, ACH_2_OCO), 4.04 (t, J = 6.5 Hz, 2H, ACH2OCO), 2.24 (s, 3H, Me), 1.86-1.79 (m, 4H, aliphatic –CH2-), 1.70-1.60 (m, 4H, aliphatic –CH2-), 1.50-1.39 (m, 16H, aliphatic –CH2-) ppm.

Figure S3. Further details on LCN preparation. a-f) Exemplificative POM images collected on cooling from the isotropic to the nematic phase for a monomeric mixture (LCN6-2) in a homogeneous planar cell. a) isotropic phase; b) formation of the nematic phase; c) and d) growth of LC domains; e) monodomain homogeneous alignment; c) monodomain homogeneous alignment rotated 45° with respect to the polarizer. h) Scheme of the steps for material polymerization.

**Table S1.** Composition and **t**hermal properties of monomer mixtures. ^a^Determined from the maximum of the transition peak in first heating (h)–cooling (c) cycle of DSC experiments (10 ºC/min). Data for the reference mixture LCN6‘-3 are reported in ref. [1].

| **Mixture** | **Monoacrylate**  **(79% mol/mol)** | **Crosslinker**  **(20% mol/mol)** | **Phase transition temperatures** [°C]^a^ | |
| --- | --- | --- | --- | --- |
| **LCN6’-6** | M6’ | CL6 | h: Cr 50 N 65 I | c: I 63 N |
| **LCN6’-8** | M6’ | CL8 | h: Cr 54 N 67 I | c: I 62 N |
| **LCN3-3** | M3 | CL3 | h: Cr_1_ 49 Cr_2_ 68 I | c: I 37 N |
| **LCN6-3** | M6 | CL3 | h: Cr_1_ 45 Cr_2_ 56 I | c: I 55 N 4 Cr |
| **LCN8-3** | M8 | CL3 | h: Cr 48 N 57 | c: I 58 N 4 Cr |

**LCN6’-8**

**LCN6’-6**

**LCN3-3**

**LCN6-3**

**LCN8-3**

**Figure S4.** DSC traces corresponding to the first cycle of heating and cooling for the monomeric mixtures (10 °C/min).

**Figure S5.** DSC second heating curves for LCN6’-3 (black line), LCN6’-6 (red line) and LCN6’-8 (blue line).

**Figure S6.** Light response of two LCN stripes (LCN6’-8 and LCN8-3’) simultaneously irradiated from the top with a green laser. The two materials bend in opposite direction at the same power and recover the original position after switching off the laser. The structure is irradiated from the opposite side with respect of that reported in Figure 6b, demonstrating as the curving direction depends only from the LC alignment and composition and not from the irradiation side.

**Video S1.** Light response of two LCN stripes (LCN6’-3 and LCN6’-8) simultaneously irradiated with a green laser at different laser powers (0.53 and 1.58 mWmm^-2^).

**Video S2.** Light response of two LCN stripes (LCN6’-8 and LCN8-3’) simultaneously irradiated with a green laser.

**References:**

[1] Martella, D.; Antonioli, D.; Nocentini, S.; Wiersma, D.S.; Galli, G.; Laus, M.; Parmeggiani, C.; Light activated non-reciprocal motion in liquid crystalline networks by designed microactuator architecture. *RSC Adv.* **2017**, *7*, 19940-19947.
